# Bipolar 1 As Graphic Memoir

**DOI:** 10.1007/s11673-016-9754-9

**Published:** 2016-12-21

**Authors:** Ellen Forney

**Affiliations:** Design Department, Cornish College of the Arts, Main Campus Center, Floor 5, 1000 Lenora St, Seattle, WA 98121 USA

**Keywords:** Bipolar, Memoir, Literature, Disability, Graphic novel

I was diagnosed bipolar shortly before my 30th birthday. Acutely manic, powerfully overconfident, and terrified that medication or even stability would kill my creativity, I refused to take meds.

When I fell into a crushing depression a few months later, I realized that no matter what happened to my art (my passion, my livelihood, my identity), my survival depended on stability. Desperate, I succumbed, and set out into the dark, tangled forest of meds, blood draws, side effects, and big learning curves.

After a years-long arc of frustrations and triumphs, recorded in stacks of sketchbooks and journals, I found a tentative stability that became increasingly reliable. I wanted to make sense of that overwhelming tangled mess, and I turned to my art to shape my experience into a graphic novel.

I’d never felt so much pressure on myself to get a story right. I needed that for my own psyche, but I also wanted to offer my story up to whoever might find something useful in it. I wanted to give specific tools I’d learned and made up, like the Cognitive Behavioural Therapy exercise I’d found helpful and a lesson on how to swallow your pills in one gulp. I wanted to give other sufferer-warriors company, as Kay Jamison (1995) and William Styron (1990) had done for me in their memoirs, *An Unquiet Mind* and *Darkness Visible*. I wanted to offer myself as a scientific case study correlating mood disorders and creativity. I wanted to transform my negative experience into something positive. I wanted it to be a good book.

**Fig. 1:**
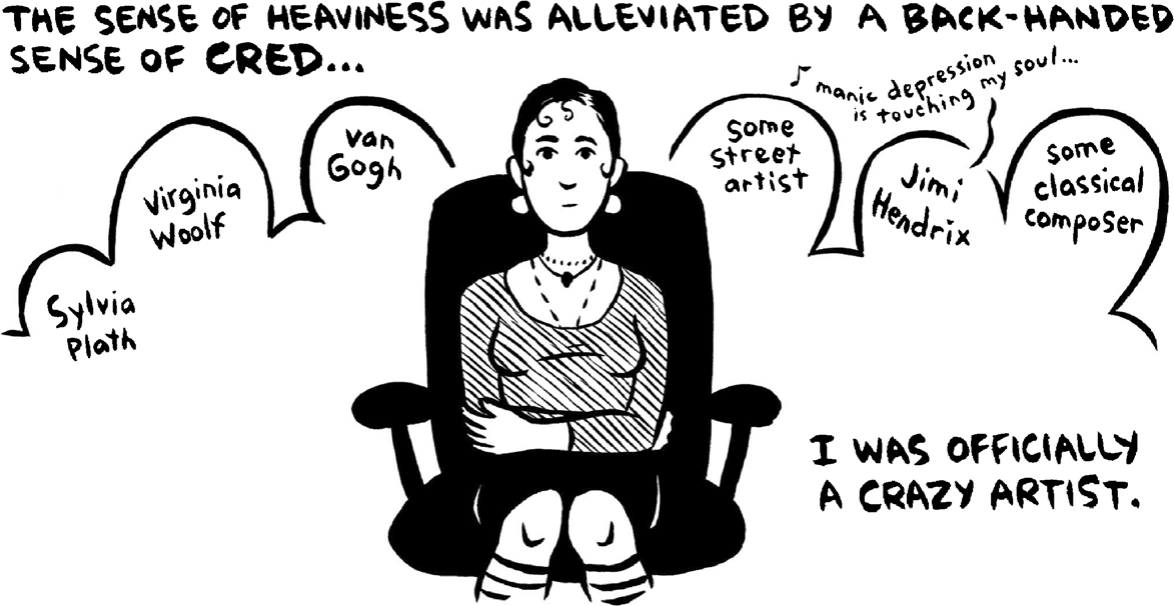
From *Marbles: Mania, Depression, Michelangelo, & Me*

In January 2012, I turned in the final draft of *Marbles: Mania, Depression, Michelangelo, and Me*. It was a strange feeling: a combination of exhaustion (Fig. 1), excitement, and tremendous anxiety. I’d always been quiet about my bipolar disorder. What would happen when people found out? Would I be forever dismissed as crazy, untrustworthy? Would people be shocked? Would it be worse if they weren’t?

I learned something huge from putting my story out in the world: as I’d hoped, people told me I was giving them company, but *I* was given so much company, too. I was not a weirdo bipolar cartoonist specimen. Strangers, friends, readers, even interviewers would *more often than not* (I mean that) disclose their own personal experience with mental illness: their own diagnosis, their family history, their friend’s suicide, their son’s struggle. I didn’t know—couldn’t have known—how many chords my story could strike or how many people were ready to be given an opportunity to come out.

Here’s the million-dollar question I get a lot: “Don’t you miss your manias?” The answer is very unsexy: “They’re not worth the risk.” No one asks if I miss my depressions!

My own, originally unexpected conclusion about being a crazy artist is that stability is good for my art. Mania was too distracting to get much work done and depression was too stifling. My current meds—lithium and lamotrigine—don’t pin me down, and a healthy lifestyle of regular sleep and good nutrition doesn’t rob me of my punk rock.

Stability is relative—I’ll always be bipolar, and I’ll always need to deal with that. My latest trick involves my blood draws, which, after all these years, I still hate: I buy myself a fancy tea drink afterwards. (My current favourite is Macha Mint Matte Soy Latte.) Now when I’m on my way to the lab, I think about my fancy tea drink. It works!

YOU HAVE COMPANY. TREAT YOURSELF NICE. And: DO YOUR ART!
